# Revolutionizing Chronic Heart Disease Management: The Role of IoT-Based Ambulatory Blood Pressure Monitoring System

**DOI:** 10.3390/diagnostics14121297

**Published:** 2024-06-19

**Authors:** Yogesh Kale, Shubhangi Rathkanthiwar, Ganesh Yenurkar, Sandip Mal, Vincent O. Nyangaresi, Shailesh Kamble, Lalit Damahe, Nandkishor Bankar

**Affiliations:** 1 Department of Electronics and Telecommunication Engineering, Yeshwantrao Chavan College of Engineering, Wanadongri, Nagpur 441110, Maharashtra, India; ycceetyogesh@gmail.com; 2 Department of Electronics Engineering, Yeshwantrao Chavan College of Engineering, Wanadongri, Nagpur 441110, Maharashtra, India; svr_1967@yahoo.com; 3 Department of Computer Technology, Yeshwantrao Chavan College of Engineering, Wanadongri, Nagpur 441110, Maharashtra, India; 4 School of Computing Science and Engineering, VIT Bhopal University, Bhopal 466114, Madhya Pradesh, India; sandip.mal@vitbhopal.ac.in; 5 Department of Computer Science and Engineering, Jaramogi Oginga Odinga University of Science & Technology, Bondo 40601, Kenya; 6 Department of Applied Electronics, Saveetha School of Engineering, SIMATS, Chennai 602105, Tamilnadu, India; 7 Department of Artificial Intelligence and Data Science, Indira Gandhi Delhi Technical University for Women, New Delhi 110006, Delhi, India; kamblesd77@gmail.com; 8 Department of Computer Science and Engineering, Yeshwantrao Chavan College of Engineering, Wanadongri, Nagpur 441110, Maharashtra, India; lalitdamahe3379@gmail.com; 9 Department of Microbiology, Jawaharlal Nehru Medical College Sawangi Meghe, Wardha 442005, Maharashtra, India; drbankarnj28@gmail.com

**Keywords:** healthcare, blood pressure, chronic heart disease, early warning score, IABPM, IoT, machine learning

## Abstract

Chronic heart disease (CHD) is a widespread and persistent health challenge that demands immediate attention. Early detection and accurate diagnosis are essential for effective treatment and management of this condition. To overcome this difficulty, we created a state-of-the-art IoT-Based Ambulatory Blood Pressure Monitoring System that provides real-time blood pressure readings, systolic, diastolic, and pulse rates at predefined intervals. This unique technology comes with a module that forecasts CHD’s early warning score. Various machine learning algorithms employed comprise Naïve Bayes, K-Nearest Neighbors (K-NN), random forest, decision tree, and Support Vector Machine (SVM). Using Naïve Bayes, the proposed model has achieved an impressive 99.44% accuracy in predicting blood pressure, a vital aspect of real-time intensive care for CHD. This IoT-based ambulatory blood pressure monitoring (IABPM) system will provide some advancement in the field of healthcare. The system overcomes the limitations of earlier BP monitoring devices, significantly reduces healthcare costs, and efficiently detects irregularities in chronic heart diseases. By implementing this system, we can take a significant step forward in improving patient outcomes and reducing the global burden of CHD. The system’s advanced features provide an accurate and reliable diagnosis that is essential for treating and managing CHD. Overall, this IoT-based ambulatory blood pressure monitoring system is an important tool for the early identification and treatment of CHD in the field of healthcare.

## 1. Introduction

Patients with chronic cardiac disease require ongoing monitoring in order to properly treat their condition, which can be difficult without the proper tools. That is why an Internet of Things-based Ambulatory Blood Pressure Monitoring System has transformed the healthcare business. This cutting-edge system gives real-time data and insights, allowing healthcare providers to provide more tailored treatment and prompt interventions. Patients can have a greater quality of life thanks to this technology, while healthcare practitioners can provide more efficient and effective care. If you want to change the way chronic heart disease is managed, an IoT-based ambulatory blood pressure monitoring system is the way to go.

Stress is a significant contributor to various health issues such as heart disease, high blood pressure, diabetes, and anxiety disorders for aging and cardiovascular disorders including hypertension and heart attack. According to research, these illnesses impact approximately 1 billion individuals throughout the world, with two-thirds of them living in poor nations [1]. In 2015, one out of every four males and one out of every five women had hypertension, and the frequency is anticipated to rise by 2022 [2]. Hypertension is one of the most prevalent cardiovascular diseases, and it dramatically raises the chance of developing Chronic Heart Disease [3]. 

Regular blood pressure monitoring is critical for reducing the risk of developing CHD and the prevalence of hypertension. For the majority of people, systolic BP drops 10% to 20% during the night [4]. This helps in the early identification and resolution of problems related to hypertension. Regular monitoring can also aid in tracking the efficacy of drugs and lifestyle modifications adopted to treat hypertension [5]. Hypertension is a global health issue since it is the biggest cause of early mortality globally. The objective is to lower the global prevalence of hypertension by 25% by 2025. Hypertension is a significant risk factor for Chronic Heart Disease [6]. By monitoring blood pressure regularly, individuals can identify and address hypertension-related concerns with early intervention, reducing their risk of developing CHD [7]. Continuous blood pressure monitoring will help discover the early warning signs and symptoms of a heart attack. For these reasons, frequent monitoring of blood pressure is required to prevent and treat cardiovascular disease. The Ambulatory Blood Pressure Monitoring System (ABPM) is a device that measures blood pressure continuously. The new ABPM provides more information on how a patient’s blood pressure variations may connect to daily activities and sleep patterns. Following the publication of new blood pressure treatment recommendations [8], using ABPM to identify hypertension.

ABPM is an advantageous method for diagnosing several stages of hypertension [9]. However, BP may rise in some circumstances, which can be detected with ABPM. ABPM can detect abnormal fluctuations in BP that may not be noticeable during clinical tests. Hypertension is considered normal when the higher blood pressure reading is less than 120 mmHg, and the lower reading is less than 80 mmHg. The BP is in the normal range, the only need is a balanced diet and regular exercise.

The Ambulatory Blood Pressure Monitoring System (ABPM) is a highly efficient technique for measuring continuous blood pressure. The ABPM is a portable device that offers extra information on a patient’s blood pressure changes as they relate to daily activity and sleep habits. It takes blood pressure measurements every 15–30 min for 24 h, offering a more thorough assessment of a patient’s blood pressure status than a single in-clinic examination. The Heart Health Alliance now recommends using ABPM for BP diagnosis since it can identify abnormal blood pressure variations that may not be seen during clinical testing. This suggestion is based on the device’s capacity to offer a more precise image of a patient’s blood pressure state, this can help in the diagnosis of hypertension and the monitoring of medication effectiveness. Table 1 below illustrates the range of different blood pressure categories.

For the purpose of early detection and treatment of hypertension-related issues, continuous blood pressure monitoring is made possible via an Internet of Things-based ambulatory blood pressure monitoring (IABPM) system.

This device continually monitors a patient’s blood pressure and makes it available via the cloud. Using this technology, healthcare providers may monitor patients’ blood pressure and offer early therapy, as necessary. This research article is primarily concerned with the development of an IABPM.

- Develop a state-of-the-art IoT-Based Ambulatory Blood Pressure Monitoring System capable of providing real-time blood pressure readings, systolic and diastolic, and pulse rate at predefined intervals.
- Utilizing advanced machine learning techniques such as Naïve Bayes, K-Nearest Neighbors (K-NN), random forest, decision tree, and Support Vector Machine (SVM) can enhance the forecasting of early warning scores for Chronic Heart Disease (CHD).
- Evaluate the accuracy of the proposed model, particularly focusing on the Naïve Bayes algorithm, in predicting blood pressure, which is critical for real-time intensive care for CHD patients.
- Assess the potential impact of the IABPM system in overcoming the limitations of earlier BP monitoring devices, reducing healthcare costs, and efficiently detecting irregularities in chronic heart diseases.
- Investigate the effectiveness of implementing the system in improving patient outcomes and reducing the global burden of CHD by providing accurate and reliable diagnosis and facilitating early detection and treatment.
- Explore the potential of the IoT-based ambulatory blood pressure monitoring system to revolutionize the field of healthcare by offering advanced features for the early detection and treatment of CHD.
- The system aims to continuously monitor blood pressure readings during daily activities, using a timer suggested by medical professionals.
- The system also designs a cloud-based platform to measure clinical data using a Wi-Fi-enabled microcontroller D1 Mini circuit, The Things Board is a platform for IoT that is open source.

The Heart Health Alliance divides hypertension, often known as high blood pressure, into four periods. These stages include elevated, hypertension stage one, hypertension stage two, and hypertension. The diagnosis is based on two blood pressure readings: systolic (higher) and diastolic (lower).

An Internet of Things-based ambulatory blood pressure monitoring (IABPM) system is a technology used to continuously monitor blood pressure. This system empowers healthcare providers to continuously monitor a patient’s blood pressure and guarantees the accessibility of the data through cloud services. Top of Form with this technology, healthcare providers can keep track of their patient’s blood pressure and provide timely interventions. To achieve this goal, we used Things Board, an open-source IoT platform, to store the data in the cloud and provide a user interface for easy analysis. The technology allows for the early prediction of chronic illnesses by establishing measurement intervals based on medical directions. This project aims to create a reliable early warning score (EWS) prediction system using sophisticated Machine learning methods like Random Forest, K-Nearest Neighbors (KNN), Decision Tree (DT), Naïve Bayes, and Support Vector Machine are utilized. The proposed system will leverage the power of these techniques to enable accurate and timely predictions of potential health emergencies, significantly reducing the risk of adverse patient outcomes. The implementation of this system will require the use of sophisticated algorithms and data analysis tools, along with a comprehensive understanding of relevant medical data and statistical methodologies. The successful development and deployment of this system will have far-reaching implications for the healthcare industry, providing healthcare professionals with a reliable and efficient tool to aid in the early detection and prevention of severe health conditions, advanced machine learning techniques can enhance the forecasting of early warning scores for Chronic Heart Disease (CHD). The research is separated into many categories. In Section 1, we introduce the International Association of Personalized Blood Management (IAPBM), which served as the foundation for our study. Section 2 presents an overview of the literature relevant to our investigation. Section 3 gives a detailed overview of our process. In Section 4, we evaluate the simulation findings and give a thorough commentary. Finally, in Section 5, we provide our conclusion and recommend further improvements.

The suggested method has the potential to transform how clinicians diagnose chronic illnesses. Medical personnel can detect chronic diseases early and intervene before they worsen by using machine learning algorithms to predict early warning scores. As a result, this study’s findings have significant implications for customized blood control and disease detection.

## 2. Related Works

The research on chronic heart disease (CHD) emphasizes its ubiquitous and urgent nature in public health. CHD is recognized as a critical global health concern, requiring immediate attention due to its extensive effect and persistent occurrence. Early detection and exact diagnosis emerge as critical strategies for the successful treatment and management of this complex illness. In response to this requirement, researchers led the creation of a novel IoT-Based Ambulatory Blood Pressure Monitoring System. This innovative device offers a paradigm leap in healthcare technology by providing real-time measures of blood pressure, including systolic and diastolic values, as well as pulse rate at predetermined intervals. The system includes a prediction module that uses machine learning methods including Naïve Bayes, K-NN, random forest, decision tree, and SVM to anticipate CHD early warning scores. The Naïve Bayes model in this system achieves 99.44% accuracy in predicting blood pressure, a crucial aspect of real-time intensive care for CHD patients. By using the capabilities of an IoT-based ambulatory blood pressure monitoring (IABPM) system, great progress has been achieved in overcoming the limitations of previous BP monitoring systems. This development not only shows promise for better patient outcomes, but it also has the potential to reduce the rising healthcare expenditures associated with CHD therapy.

Furthermore, the system’s ability to detect anomalies in chronic cardiac disorders is a significant development in cardiovascular health. By adopting this innovative technology, healthcare practitioners may usher in a new era of precision medicine, providing precise and reliable diagnoses critical for the comprehensive treatment and management of CHD. The IoT-based ambulatory blood pressure monitoring system appears as a transformational tool in the early identification and treatment of CHD, with the potential to overhaul the healthcare environment. Its use in clinical practice marks a huge step forward in tackling the worldwide burden of CHD and Enhancing patient care and results.

The utilization of IoT in medical science has been extensive, particularly in monitoring patients’ health. The following studies are interconnected with this theme. Tamilselvan and colleagues developed a device for IoT-based health monitoring, which operates on a web platform and tracks Vital indications such as oxygen saturation percentage, heart rate (HR) in beats per minute (bpm), eye movement, and patient body temperature. The device comprises an eye blink sensor, an Sp-O2 sensor for oxygen saturation, and a thermometer. Data processing is conducted on an Arduino-UNO board within an existing IoT-based health monitoring system. However, no specific performance trials for any patients are documented, as indicated in Table 2 [10]. Acharya introduced an IoT healthcare monitoring kit. This system oversees various crucial health parameters, including respiration, body temperature, ECG, and heartbeat. The research employed an ECG sensor on the Raspberry Pi along with blood and HR (bpm) monitoring. Regardless of the clinical data measured, initial processing occurs on a Raspberry processor before being transmitted to the IoT network. The study concludes that the most significant issue with the system is the lack of adequate interfaces for data visualization [11].

Gregoski, the researcher, initially proposed utilizing smartphones to display an individual’s heart rate (bpm). By utilizing the mobile’s brightness and camera, the study tracked changes in blood flow, assessing the cardiac signal accordingly. The result of this innovative technology allows users to monitor their heart rate simply by observing their phones, eliminating the need for manual input. However, the researcher emphasizes that if continuous pulse monitoring throughout the day without hindering the patient’s movement is necessary, the suggested method is ineffective [12]. Researchers Oresko JJ. et al. have developed two CVD detection platforms that are wearable and compatible with smartphones. These pioneering platforms facilitate the acquisition, display, extraction of features, and classification of beats in real-time electrocardiograms. The significance of these platforms lies in their potential to detect CVD in a timely manner, thereby improving patient outcomes and reducing healthcare costs. The real-time ECG acquisition functionality allows for continuous monitoring of patients’ heart health, which is especially useful for those at risk of CVD. The feature extraction and beat classification capabilities provide valuable insights into the condition of the heart and enable healthcare professionals to make informed decisions about the appropriate course of treatment. Overall, these platforms represent a significant step forward in the detection and management of CVD, and their potential to improve patient outcomes is truly exciting. Alternatively, implementing the same technique using cell phones is feasible if cost and time are not a concern. Oresko suggests utilizing this technique for detecting cardiovascular illnesses. However, the study concludes that while the proposed prototype successfully observes coronary rhythm in real-time, it struggles to track heart rate (bpm) over time, making it challenging to detect cardiovascular diseases [13].

While S. Trivedi et al. [14] have developed an Android-based health parameter monitoring system, sensors are used to non-invasively measure parameters. The most recent and popular healthcare technology, which includes a smartphone and an Arduino-based system. Utilizing this technology, medical practitioners may remotely monitor and evaluate clinical data as well as prescribe medications. Kumar [15] also developed a tailored safety nursing gadget utilizing IoT. It is divided into three sections: control, gadget, and convince. By strategically implementing cutting-edge machine learning techniques, the accuracy of early warning scores for Chronic Heart Disease (CHD) might be greatly improved in the field of early detection and prevention of severe health issues. We have effectively utilized the IC-DS18B20 temperature sensor in conjunction with a pulse sensor to enable the diligent monitoring of patients’ vital signs, such as temperature and pulse rate. Using a variety of devices, including laptops, desktop computers, and mobile phones, healthcare workers can now quickly access and evaluate critical information thanks to the smooth transfer of clinical data from Arduino to a cloud-based platform. Because they had access to a full dataset, they were now better equipped to make educated decisions and implement customized therapies.

Desai’s research [16,17] involved the use of a wireless sensor network (WSN) to monitor patients’ heartbeats. The researcher employed Spartan3 to process parallel data on an FPGA. Nonetheless, the work was limited in three main ways. Firstly, the data was not available on a cloud platform. Secondly, there was no timer facility. Thirdly, the wireless sensor network was characterized by high power consumption [16,17].

New techniques for optimizing data preprocessing have been introduced by Puneet Mishra et al. [18] who have described various approaches, including scatter correction techniques, which provide complementary information. Ensemble fusion is used to improve chemometrics models by utilizing this complementary information, with ensemble techniques based on multi-block data analysis outperforming others.

Fast data exploration and prototyping are made possible by the Seaborn package, created by M. L. Waskom [19], which offers an interface to Matplotlib and preserves the stability and flexibility needed for publication-quality graphics. Viewing a broad variety of well-represented tabular datasets is possible with its domain generality. The text you wanted me to keep in mind is as follows:

Machine learning techniques for analyzing medical datasets have been reviewed by D. Bhende et al. [20]. The Arduino Uno microcontroller board is connected to these sensors, which then transmit data to the user’s Android smartphone via Bluetooth. Meanwhile, Ogunpola A. et al. [21] have explored the early detection of heart diseases, specifically myocardial infarction, through machine learning techniques. The primary objectives are to boost patient outcomes, lessen the load on healthcare systems, and improve early detection and prognosis.

To address these limitations, the proposed research introduces the Blynk IoT and Things Board application to enable the availability of clinical data on the cloud. Additionally, the research enables the Ambulatory Blood Pressure Monitoring System ABPM no-timer facility by introducing a transistor circuit and low-power circuitry. Table 2 shows that the comparative analysis of the previous techniques related to heart disease prediction.

Researchers have made significant strides in understanding and combating sepsis, a potentially life-threatening condition. Albur M. et al. [22] have devised a dynamic indicator of severity and prognosis for the Early Warning Score in patients with Gram-negative bacteraemia and sepsis. Similarly, Ng R. et al. [23] have constructed and validated prognostic models tailored to different sexes, exhibiting robust predictive performance, discrimination, and calibration across developmental, internal, and external validation phases. Siddiqui E. et al. [24] have illustrated that the Early Warning Score (EWS) exhibits high sensitivity and specificity in identifying sepsis, severe sepsis, and septic shock, thereby serving as a valuable tool for early diagnosis and effective management of these conditions.

Khekare, Ganesh, et al. [25] explored the capabilities, attributes, and challenges associated with the Internet of Things (IoT). They have traced the path of IoT from its inception to its impending impact on people’s quality of life worldwide. The IoT has tremendous potential to enhance the efficiency and convenience of various aspects of human life, but its implementation also presents significant challenges. Overall, these studies contribute to our understanding of sepsis and IoT and pave the way for further research in these critical areas.

## 3. Materials and Methods

### 3.1. Overview of IAPBM

Revolutionizing Chronic Heart Disease Management: The Role of IoT-Based Ambulatory Blood Pressure Monitoring Systems is likely to discuss how emerging technologies, specifically the Internet of Things (IoT), are changing the way chronic heart disease is managed, particularly through the implementation of ambulatory blood pressure monitors. Here is the overview of the IABPM system provided below:

❖ About Chronic Heart Disease (CHD):
  ▪ Cardiovascular disease (CHD), its prevalence, and its impact on public health.
  ▪ Emphasize the significance of good management options for CHD patients to reduce complications and enhance outcomes.
❖ Current Challenges in CHD Management:
  ▪ Explore the limits of established approaches for monitoring CHD patients, such as clinic visits and blood pressure monitoring devices.
  ▪ Emphasize the need for continuous and remote monitoring to enable prompt interventions and individualized care.
❖ Ambulatory Blood Pressure Monitoring (ABPM):
  ▪ Define ABPM, which entails continuously measuring blood pressure in a patient’s natural surroundings for 24 h.
  ▪ Discuss the benefits of ABPM over traditional blood pressure monitoring, such as the capacity to identify nocturnal hypertension, white coat hypertension, and masked hypertension.
❖ Role of IoT in CHD Management:
  ▪ Discuss IoT technologies and their potential use in healthcare, such as remote patient monitoring.
  ▪ Discuss how IoT-enabled ABPM devices may capture real-time data from CHD patients and send it to healthcare practitioners for analysis and interventions.
❖ Features and Components of IoT-Based ABPM Systems:
  ▪ Describe IoT-based ABPM system components, such as wearable blood pressure monitors, data transmission protocols, and cloud-based storage and analytics platforms.
  ▪ Explore system features including automated data synchronization, real-time notifications for aberrant readings, and user-friendly interfaces for patients.
❖ Benefits of IoT-Based ABPM Systems:
  ▪ Use IoT-based ABPM systems for CHD management to increase patient adherence, detect hypertension problems early, and optimize healthcare resources.
  ▪ Provide data from research or clinical trials to demonstrate the usefulness of these systems in improving patient outcomes.

An Internet of Things-based Ambulatory Blood Pressure Monitoring System can transform chronic heart disease care by offering continuous, individualized monitoring, early identification of irregularities, and initiative-taking intervention to enhance patient outcomes and quality of life.

### 3.2. Combine System Architecture

**Figure 1:**
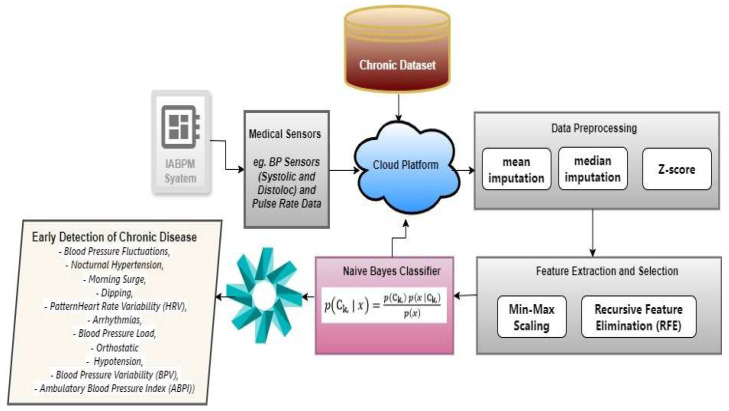
Combine system diagram.

The IABPM is a phenomenally successful medical instrument that was methodically designed and evaluated at Shalini Tai Meghe Hospital in Nagpur, India. This innovative instrument is specially created to address heart-related chronic disorders such as normal, raised, high-BP hypertension stage 1 and stage 2. By emphasizing the significance of obtaining early medical attention in hypertension emergencies, the IABPM enables people to take care of their health and manage their condition proactively. With an established record of performance, the IABPM is a game changer in hypertension care, set to alter how we manage this crucial health issue. The following Figure 1 shows the combined system diagram for IABPM including hardware and software modules.

Figure 1 illustrates a complex Integrated Automated Business Process Management (IABPM) system that comprises several carefully designed stages, such as dataset collection, IABPM system, and model generation. Every stage is interrelated and essential to the overall operation of the system. The processes are carried out in a specified order to achieve the greatest possible results, and the entire system is meticulously planned to give maximum efficiency and efficacy.

#### 3.2.1. Hardware Specification of IABPM System

**Figure 2:**
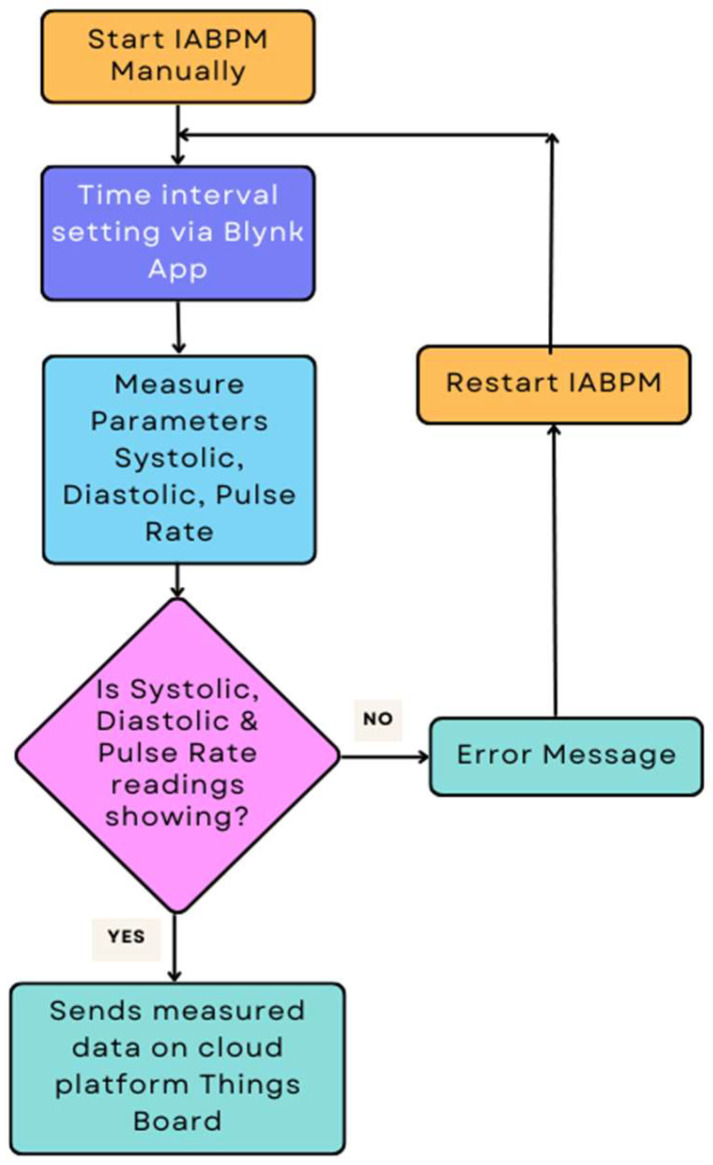
IABPM flow diagram [26].

Given the system’s ability to gather and transmit data, the IABPM system has the capacity to transform the approach healthcare professionals use to diagnose and treat patients. By collecting and transmitting vital data, the IABPM system provides medical professionals with a valuable tool to enhance patient care outcomes. Figure 2 depicts the workflow of the IABPM system.

**Figure 3:**
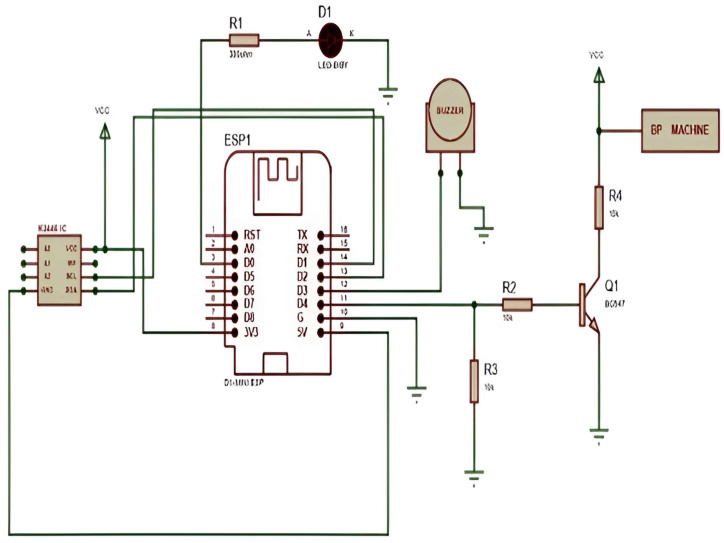
Circuit diagram of an IABPM [26].

The diagram depicted in Figure 3 illustrates the distinctive circuit design of the IABPM system, which is fundamental in elucidating its operation. The IABPM system is engineered to activate and deactivate the medical sensor device per a predetermined time interval specified by medical experts. The medical sensor device is furnished with the capability to record and transmit data related to blood pressure and pulse rate, which is subsequently conveyed to the cloud for further analysis by healthcare professionals.

This feature-rich system is designed to conserve power by preventing the medical sensor device from consuming power unnecessarily, thereby prolonging the device’s battery life. The IABPM system’s medical sensor device serves as a critical source of information, providing clinicians with pertinent data that can help them make informed decisions about patient care.

A model that has been trained employs machine learning algorithms such as Naïve Bayes, KNN, Random Forest, Decision Tree, and SVM for data pre-processing. Using pre-processed data, the algorithm produces early warning scores (EWS) and forecasts chronic illnesses. This proposed approach is useful for remote patient monitoring, allowing medical practitioners to spot possible health risks in a timely way.

The Intelligent Automatic Blood Pressure Monitor (IABPM) system comprises a D1 microcontroller, a switching circuit based on transistors, and a blood pressure sensor. The system’s core component is the D1-mini controller, which includes a Wi-Fi module as well as a microprocessor. The blood pressure sensor is actuated by the D1-mini controller, which is controlled via a mobile app.

The IABPM system is a highly advanced and sophisticated technology that is designed to provide accurate and reliable readings of blood pressure levels. The D1 microcontroller is a powerful and efficient device that is capable of performing complex functions and computations. The transistor-based switching circuit is an essential component of the system that enables the D1 microcontroller to control the blood pressure sensor.

Furthermore, the IABPM system utilizes a mobile application that allows users to easily operate and control the device. The mobile application is user-friendly and intuitive, which makes it easy for users to access and interpret the data provided by the system. Overall, the IABPM system is a revolutionary technology that is poised to revolutionize the way blood pressure levels are monitored and measured.

The Blynk mobile application has an ON/OFF BP sensor with a timer that allows users to take readings at specific intervals. The BP-02 sensor also sends clinical data to the D1-mini controller, which is available via the Things Board cloud platform. The circuitry comprises a driver IC, a D1-mini controller, 330-ohm resistors, LED, and buzzer, and a NPN transistor for switching the BP sensor.

- Functioning of IABPM Device:

- *Devices and Sensor*: High-precision sensors that detect pulse rate and systolic and diastolic blood pressure are found in wearable and portable monitors. Connectivity: Devices that can transmit data via Bluetooth or Wi-Fi modules.
- *Internet of Things Connectivity*: Data Transmission: Real-time data transport from the monitors to a central server or cloud-based infrastructure is guaranteed via wireless communication protocols, such as Bluetooth and Wi-Fi.
- *Data Processing Unit*: Central Server/Cloud: Gathers information from various sources, preprocesses it, and saves it for further examination.
- *User Interface*: Mobile App/Web Portal: Patients and healthcare providers may access historical trends, real-time data, and alarms by using the Blynk mobile application.

b. Measuring Data from the IABPM

The recorded data can be saved in the device’s Electrically Erasable Programmable Read Only Memory and retrieved by the D1-mini controller utilizing the Inter-Integrated Circuit (I2C) protocol after a measurement. The user can trigger the data periodically, as advised by medical professionals, to ensure that the data is up-to-date and accurate. After the measurement, the stored data can be retrieved from the EEPROM using the I2C protocol.

The serial data line (SDA) is connected to the D2-pin, and the serial clock line (SCL) pin is used to synchronize data transfers through the EEPROM’s I2C bus. Additionally, the D3-pin is linked to a buzzer that generates an audible sound when the measurement is completed, signifying the end of the procedure. This feature can be particularly beneficial in situations where the user is occupied with other tasks.

#### 3.2.2. Uploading Clinical Data on the Cloud Platform

The clinical data, which includes blood pressure (BP) and heart rate (HR) readings, can be easily retrieved and uploaded to Things Board, an open-source platform that provides an array of widgets, charts, and graphs that can be incredibly useful for both medical professionals and patients. This system allows for the monitoring of clinical data through various Information and Communication Technologies (ICT), such as mobile phones, laptops, or personal computers, making the process more convenient and accessible.

The BP readings are measured using an IABPM, which provides precise systolic measurements of 124 mmHg, diastolic measurements of 93 mmHg, and pulse rate readings of 77 bpm. Once the data is measured, it is stored on the cloud through the Things Board, allowing for secure and easy access to the clinical data for both patients and medical professionals. The data showed varying systolic and diastolic values across five categories as mentioned in Table 3.

The patient uses the Blynk mobile app to manually activate the IABPM system and adjust the time interval. The blood pressure sensor gauges systolic, diastolic, and pulse frequencies. The system checks the correctness of the readings. If the readings are accurate, they will be sent immediately to Things Board, an open-source IoT platform. In the event of an error, the system will restart and repeat the operation until the right readings are presented.

#### 3.2.3. Data Preprocessing

Pre-processing stands as a crucial phase within machine learning algorithms, encompassing the preparation and transformation of datasets to enhance the process of knowledge discovery. When working with medical data sets, each patient has unique attributes with comparable values, making data normalization for attributes impossible. As a result, the Entry_id and Target properties were deleted during pre-processing to guarantee proper analysis. The proposed IABPM pre-processed the dataset by incorporating the following techniques: The aggregation of the data made available through vital signs and historical health data.

- *Vital Signs*: Continuous real-time measurements of heart rate and blood pressure are taken.
- *Historical Health Data*: Combining information from Electronic Health Records (EHR) with patient history, lifestyle choices, past episodes of CHD, and other relevant data.

- Mean and Median Imputation

Missing values are a prevalent concern in Cardiovascular Heart Disease (CHD) data processing, reducing the accuracy of the analysis and modeling process. As a result, addressing these data gaps is critical to obtaining accurate and trustworthy results. To that purpose, mean and median imputation approaches have become extensively used as easy and effective means of filling in missing information. Researchers who use these strategies can improve the quality and dependability of their analysis and modeling results.

Mean imputation is simple and may be applied with numerical variables. It assumes missing values occur at random and replaces them with the average value of the available data (Equation (1)). This strategy retains the dataset’s overall mean.

(1) $$\bar{x}=\frac{a+\Sigma fixi}{\Sigma fi},$$

where a is the Assumed mean, and di is equal to xi— a ∑fi the sum of all frequencies.

Median imputation is identical to mean imputation except that the median is used instead of the mean. It is less sensitive to outliers than mean imputation and is frequently used when data contains extreme values (Equation (2)).

(2) $$Median=l+\left[\frac{n}{2}-cf\right)/f]*h].$$

In this equation, “ l ” denotes the lower limit of the median class, “ n ” stands for the total count of observations, “ f ” indicates the frequency of the median class, “ h ” represents the class interval, and “ cf ” signifies the cumulative frequency of the class just before the median class.

b. Z-Score

The z-score (Equation (3)), commonly referred to as the standard score, is a statistical measure that indicates the extent to which a data point differs from the average of the dataset. Its computation is based on the formula provided below.

(3) $$z=\frac{X-\mu }{\sigma },$$

where:

-
x represents the value of the data point.
-
μ is the dataset’s meaning.
-
σ represents the dataset’s standard deviation.
- z represents the z-score.

The z-score is an accurate approach for comparing individual data points to the dataset’s mean. A positive z-score signifies that a data point surpasses the mean, whereas a negative z-score indicates it falls below the mean. The magnitude of the z-score reflects the extent of deviation from the mean. Using these preprocessing techniques, data from the IoT-Based Ambulatory Blood Pressure Monitoring System can be properly prepared for training machine learning models, resulting in more accurate predictions of CHD’s early warning score and, ultimately, better patient outcomes in the detection and management of chronic heart diseases.

#### 3.2.4. Extracting and Selecting Features

Feature extraction and selection are key components of predictive model construction, especially for healthcare applications like the IoT-Based Ambulatory Blood Pressure Monitoring (IABPM) System you described in your article. Here is an example of how this system’s feature extraction and selection may be approached: Feature extraction is converting raw data into a set of relevant features that may be used as inputs to prediction models. The raw data from the IABPM system is expected to comprise real-time blood pressure readings (systolic and diastolic) and pulse rate recorded at specified intervals.

- Min-Max Scaling

Min-max scaling is a prominent preprocessing strategy in machine learning that rescales features to a given range, usually between 0 and 1. This normalization procedure guarantees that all features have the same scale, preventing characteristics of greater size from dominating the model’s learning process. Here’s how min-max scaling may be used to improve the functionality of the IoT-Based Ambulatory Blood Pressure Monitoring System. For each feature X , apply the min-max scaling transformation using the following formula (Equation (4)):

(4) $$X_{scaled}=\frac{X-X_{min}}{X_{max}-X_{min}}$$

-
X _scaled_ represents the rescaled value of feature X .
-
X _min_ denotes the minimum value observed for feature X .
-
X _max_ signifies the maximum value observed for feature X .

b. Recursive Feature Elimination

The aim of feature selection is to identify the most relevant set of attributes that enhance the predictive accuracy of the model, all the while reducing complexity and guarding against overfitting. Given the potentially substantial number of extracted features from the IABPM system, feature selection becomes crucial for model interpretability and computational efficiency.

Applying Recursive Feature Elimination to the information from the IoT-Based Ambulatory Blood Pressure Monitoring System allows you to select the most significant characteristics for forecasting CHD’s early warning score, possibly enhancing the predictive models’ accuracy and interpretability. This feature selection technique can minimize the dataset’s dimensionality and focus on the most important characteristics, resulting in more efficient and effective identification and management of chronic heart disease.

Extracting and choosing characteristics is critical in creating precise and interpretable prediction models for healthcare applications like the IoT-Based Ambulatory Blood Pressure Monitoring System. Construct a prediction model that reliably detects abnormalities in chronic heart disease by detecting the most significant properties in raw data and choosing the most relevant subset. This allows for early detection and treatment, leading to better patient outcomes. This transformational strategy has the potential to disrupt the healthcare profession by offering patients more effective care and treatment options.

#### 3.2.5. Model Selection

Once the relevant features have been extracted and selected, they can be used as inputs to machine learning algorithms for developing predictive models to predict CHD’s early warning score. Choosing the best learning algorithm for a certain job, such as transforming chronic heart disease treatment with an IoT-based ambulatory blood pressure monitoring system, necessitates careful evaluation of several criteria.

This study uses a Naïve Bayes classifier function (Equation (5)) to analyze abnormalities. This model is commonly used by IABPM systems and is intended to assign a probability ( P) for each of the K potential outcomes or classes (C_k_) with regard to a specific issue instance requiring categorization. An instance is represented as a vector (x) that encodes an independent variables or characteristics (x1, x2, …, xn). The study’s goal, using this methodology, is to diagnose abnormality concerns precisely and confidently.

(5) $$p\left(C_{k}|x\right)=\frac{p{(C}_{k,})p\left(x\right|C_{k,})}{p(x)}.$$

Applying the chain rule to ABPM of conditional probability, we can express Equation (2) in a more simplified manner. This approach involves breaking down a complex probability into smaller, more manageable components, resulting in a more efficient calculation process. By using this method, the relationship between the conditional probabilities participates in Equation (6).

(6) $$\begin{array}{cc} p\left(C_{k,}x_{1,\ldots .}x_{n}\right) & =p\left(x_{1,\ldots .}x_{n,}C_{k}\right)=p\left(x_{1}|x_{2,\ldots .}x_{n,}C_{k}\right)\;p\left(x_{2,\ldots .}x_{n,}C_{k}\right)\\ & =p\left(x_{1}|x_{2,\ldots .}x_{n,}C_{k}\right)\;p\left(x_{2}|{x_{3,\ldots .,}x}_{n,}C_{k}\right)p\left(x_{3,\ldots .}x_{n,}C_{k}\right)\\ & =p\left(x_{1}|x_{2,\ldots .}x_{n,}C_{k}\right)\;p\left(x_{2}|{x_{3,\ldots .,}x}_{n,}C_{k}\right)\ldots p\left(x_{n-1}|x_{n,}C_{k}\right)p\left(x_{n}|C_{k}\right)p\left(C_{k}\right). \end{array}$$

Assuming a conditional category, x serves as a mutually independent variable (Equation (7)).

(7) $$p\left(x_{i\;|\;,}x_{i+1,\;\ldots ,}x_{nC_{k}}\right)=p\left(x_{n|}C_{k}\right)$$

Thus, the joint model may be expressed as follows (Equation (8)):

(8) $$\begin{array}{cc} P\left(C_{i\;|\;}x_{1,\ldots ,}x_{n}\right) & =\propto p\left(C_{k,x1,\ldots }x_{n}\right)\\ & \;\propto p\left(C_{k}\right)P\left(x_{1\;|\;}C_{k}\right)P\left(C_{x2\;|\;}C_{k}\right)\;P\left(C_{x3\;|\;}C_{k}\right)\ldots \\ & \;\;\;\;\;\;\;\propto p\left(C_{k}\right)\prod_{i=1}^{n}P\left(C_{xi\;|\;}C_{k}\right), \end{array}$$

where proportionality is denoted by ∝. Equation (9) illustrates the distribution conditioned on the class variable C , given the independence assumptions stated above.

(9) $$P\left(C_{k\;|\;}x_{1,\ldots ,}x_{n}\right)=\frac{1}{Z}p\left(C_{k}\right)\;\prod_{i=1}^{n}P\left(C_{xi\;|\;}C_{k}\right)$$

Once the feature variable values are determined, the evidence $Z=p\left(x\right)=\sum_{k}p\left(Ck\right)p\left(x\right|Ck)$ is calculated. This is a scaling factor that depends entirely on the values of x1, x2, …, xn.

(10) $$ŷ=\underset{\mathrm{k\epsilon}\{1,\ldots ,k\}}{\mathrm{argmax}}p\left(C_{k}\right)\;\prod_{i=1}^{n}P\left(C_{xi\;|\;}C_{k}\right)$$

To reduce misclassification, the model is linked with the maximum a posteriori decision rule, which is based on the Naïve Bayes classifier. This rule recommends choosing the most likely hypothesis to minimize misclassification. The Bayes classifier uses Equation (10) to categorize k.

#### 3.2.6. Clinical Trials and Validation Processes

The validation process is carried out by clinical trials, benchmarking against standard devices and validation of algorithms.

- *Clinical Trials*

Initial small-scale trials with 20–30 participants to test system functionality and preliminary efficacy.

- *Benchmarking Against Standard Devices*

Blood pressure readings from the IABPM system are compared with those from clinically validated monitors.

- *Algorithm Validation*

It can be done through k-fold cross-validation to evaluate and compare the performance of each machine-learning model. And the performance metrics like Assessing accuracy, precision, recall, F1-score, and ROC-AUC to ensure model reliability.

The IoT-Based Ambulatory Blood Pressure Monitoring System operates by continuously collecting and transmitting real-time data, which is processed and analyzed using advanced machine learning models. The system calculates an early warning score for CHD by aggregating real-time monitoring data with historical health information, validated through rigorous clinical trials and benchmarking processes. This comprehensive approach ensures accurate, reliable, and actionable insights for the early identification and management of chronic heart disease.

#### 3.2.7. Security and Privacy of Patient’s Data

The IoT-Based Ambulatory Blood Pressure Monitoring System incorporates robust data encryption and security measures to safeguard patient privacy and prevent unauthorized access. Following are the ways to secure the patients data:

- *End-to-End Encryption*: Robust cryptographic techniques should be used to encrypt all data transferred between the central database and the monitoring device. This makes sure that the data is unintelligible by unauthorized persons even if it is intercepted.
- *Secure Authentication*: To limit access to the system, utilize secure authentication techniques like biometric authentication or username/password combinations. This guarantees that patient data can only be accessed by authorized healthcare personnel.
- *Physical Security*: Make sure there are safeguards in place to prevent theft or tampering with the IoT devices themselves. Tamper-evident seals, secure enclosures, and location-based access restrictions are a few examples of this.
- *Firmware and Software Updates*: Maintain the most recent security updates installed on the firmware and software of your Internet of Things devices to minimize vulnerabilities and guarantee ongoing defense against emerging threats.

Through the use of data encryption and security measures, the Internet of Things-Based Ambulatory Blood Pressure Monitoring System may successfully safeguard patient privacy and thwart illegal access, guaranteeing the integrity and confidentiality of private medical data.

## 4. Experiment Results and Discussion

The section presents an innovative IoT-Based Ambulatory Blood Pressure Monitoring System designed to tackle the challenges associated with early detection and precise diagnosis of Chronic Heart Disease (CHD). This system provides real-time readings of blood pressure, systolic and diastolic, and pulse rate at predefined intervals, enhancing the capability for continuous monitoring and timely intervention.

The importance of early detection and accurate diagnosis in managing CHD highlights the transformative impact of the IoT-based ambulatory blood pressure monitoring system in revolutionizing healthcare delivery.

- *Initial Setup*: To ensure that the monitoring system is properly set up and all components are functioning correctly.
- *Standardization of Equipment*: Ensure that this reference device is regularly calibrated to maintain accuracy.
- *Preparation of Calibration Environment*: To select a quiet and stable environment free from disturbances or vibrations that could affect the accuracy of the calibration process.
- *Zero Calibration*: To ensure the blood pressure cuff is properly zeroed before calibration. This involves verifying that the cuff reads zero pressure when not applied to a patient.
- *Pressure Calibration*: To apply known pressures to the blood pressure cuff using the reference standard device. Gradually increase the pressure in increments while recording the corresponding readings from both the reference standard and the IoT-based monitoring system.
- *Comparison and Adjustment*: To compare the readings obtained from the IoT-based monitoring system with those from the reference standard. If there are any discrepancies, adjust the calibration settings of the IoT system accordingly.
- *Validation*: After adjusting the calibration settings, repeat the calibration process to validate the accuracy of the adjustments made. Continue this iterative process until the measurements obtained from the IoT-based system align closely with those from the reference standard.
- *Verification*: Once calibration is complete and validated, perform periodic verification checks to ensure the continued accuracy of the blood pressure cuff. This may involve recalibration at regular intervals or whenever there are significant changes to the system.
- *Documentation*: Document the calibration process, including the calibration settings applied and any adjustments made. Maintain records of calibration activities for auditing and quality assurance purposes.

The IoT-Based Ambulatory Blood Pressure Monitoring System can be made more reliable for the early identification and treatment of chronic heart disease by calibrating the blood pressure cuff according to this calibration technique, which will guarantee accurate and consistent readings.

### 4.1. IABPM System Plan

It is vital to carefully evaluate scalability, implementation, and necessary technical modifications in order to expand the scope of the IoT-Based Ambulatory Blood Pressure Monitoring System to address other chronic conditions. The following is a thorough explanation of how to do this.

- *Finding Similar Chronic Diseases*: Keep an eye out for problems including hypertension, diabetes, chronic kidney disease, and some respiratory disorders like COPD that require similar levels of monitoring to chronic heart disease (CHD).
- *Scalability considerations*: Make sure the system architecture has modular components that can be used to monitor multiple physiological indicators associated with chronic diseases and facilitate the integration of extra sensors and data streams as required for distinct scenarios.
- *Gathering and analyzing data*: It is critical to create machine learning models and algorithms tailored to particular diseases. The system will assess blood pressure and glucose levels for diabetic management; it will monitor breathing rate and oxygen saturation for COPD. To enable precise prediction and early anomaly identification, these models will be trained on datasets relevant to particular diseases.
- *Electronic Health Records (EHR) Integration*: Easily integrate with EHR systems to give medical professionals instant access to a wealth of patient data, facilitating comprehensive patient management and individualized treatment plans.
- *User Interface and Accessibility*: Improve the user interface to accommodate various chronic illnesses and enable patients and healthcare providers with configurable dashboards that easily and clearly display pertinent health measurements, trends, and alarms.
- *Remote Monitoring and Telemedicine*: Use telemedicine for virtual consultations and remote monitoring of patients with chronic diseases. This is particularly helpful for people who live in rural places or have limited mobility.

#### 4.1.1. Implementation Plan

Step 1: To perform a thorough requirements analysis and a scalability feasibility assessment.
Step 2: To adapt the current system architecture to incorporate disease-specific algorithms that have been developed.
Step 3: To examine the enhanced system in a clinical setting with patients who have a range of chronic illnesses.
Step 4: To get input from patients and healthcare professionals to improve the usability and efficacy of the system.
Step 5: To implement the IoT-based monitoring system that is scalable in all healthcare facilities. Implementation should start small and work its way up to larger populations.

#### 4.1.2. Technical Improvements

- *Improved Sensor Technologies*: Make an investment in state-of-the-art sensor technologies that can measure a greater variety of physiological characteristics accurately and precisely.
- *Sustained Performance Monitoring*: Establish systems for continuous performance monitoring and feedback loops to guarantee the system’s accuracy and long-term dependability.
- *Procedures for Security and Privacy*: To protect patient data and guarantee adherence to healthcare laws like GDPR (General Data Protection Regulation) and HIPAA (Health Insurance Portability and Accountability Act), and strengthen security measures.

By adopting an all-encompassing strategy, the Internet of Things-based Ambulatory Blood Pressure Monitoring System can be efficiently expanded to handle a wider range of chronic illnesses, leading to better patient outcomes and a decrease in the overall strain on healthcare systems.

### 4.2. About Dataset: Collection of Patients Sample Data

This research explores three methods for collecting clinical data in healthcare. The first method involves a BP sensor collecting clinical data in a real-time manner for any computer system. The second method involves blind manual readings using standard ward devices, and the third method combines both, allowing medical professionals to add clinical notes. A large dataset of 600 clinical samples has been collected from Shalini Tai Meghe Hospital Research Centre for this research work.

Table 4 presents a variety of clinical data from the SMHRCE hospital, providing a comprehensive overview of 600 samples with eight features. The data come from the proposed IABPM system, which ensures its correctness and dependability. The data is organized in the table by entry id, patient age, systolic, diastolic, pulse rate, patient gender, chronic illness degree, and target attribute, allowing for a full examination of the patient’s health state. The data will be largely utilized for result analysis and future research, which will aid in improving the patient care process and the overall quality of healthcare delivery.

### 4.3. IABPM Evaluation Metrics

To evaluate the effectiveness of an Ambulatory Blood Pressure Monitoring System based on IoT in detecting and managing chronic heart disease, it is crucial to take into account metrics that measure the system’s accuracy and ability to provide timely insights into the cardiovascular health of patients. Here are some important metrics that need to be considered for assessment:

#### 4.3.1. Accuracy

Accuracy is a measurement of how accurate a system’s predictions are. It is determined by dividing the number of accurately anticipated cases by the total number of instances. However, relying merely on accuracy may be insufficient if the dataset is skewed or false positives/negatives have different effects (Equation (11)).

(11) $$Accuracy=\frac{Number\;of\;Corrrect\;predictions}{Total\;no.\;of\;predictions}\times 100.$$

#### 4.3.2. Precision

Precision is a measure evaluating the accuracy of a system in correctly identifying positive instances, like patients with CHD, among all instances it categorizes as positive. To obtain this figure, divide the count of true positives by the sum of true positives and false positives. (Refer to Equation (12) for details).

Precision = T_P/(T_P + F_P).(12)

#### 4.3.3. Recall (Sensitivity)

By calculating the proportion of true positives relative to the sum of true positives and false negatives, recall evaluates how effectively a system can correctly identify all positive instances within a dataset. (Refer to Equation (13) for details.)

Recall = T_P/(T_P + F_N).(13)

#### 4.3.4. F1-Score

The F1-score, a unified metric, effectively balances both precision and recall, proving particularly valuable in cases of class imbalance. It is calculated as the harmonic mean of precision and recall. (Refer to Equation (14) for details).

F1-score = 2 × (Precision × Recall)/(Precision + Recall).(14)

#### 4.3.5. Area under the Receiver Operating Characteristic Curve (AUC-ROC)

ROC curves illustrate how well a classification model performs across various thresholds. The AUC-ROC reflects the model’s capability to differentiate between positives and negatives.

#### 4.3.6. Mean Absolute Error (MAE) or Root Mean Squared Error (RMSE)

If the system is predicting continuous variables (for example, blood pressure measurements), MAE or RMSE can be used to calculate the Average gap between projected and actual values. Lower values correspond to higher performance.

The percentage of variation in the independent variable is symbolized by R ^2^, which is a linear model and is expressed in Equation (15).

(15) $$R^{2}=\frac{Variance\;explined\;by\;model}{Total\;variance}.$$

A refined version of R ^2^ is the Adjusted R-squared ( R ^2^ adjusted), which, similar to R ^2^, indicates the goodness of fit of the data points to the curve. This is defined in Equation (16).

(16) $$R^{2}\;adjusted=1-(1-R^{2})\frac{n-1}{n-(k+1)}.$$

In this case, K indicates the number of independent variables in the regression equation, and n specifies the sample size. As specified in Equation (17), mean absolute error ( MAE ) refers to the average size of mistakes in model predictions.

(17) $$MAE=\frac{1}{n}\sum_{j=1}^{n}\left|y_{j}-{\hat{y}}_{j}\right|.$$

Mean square error ( MSE ) is the distance of data points from the regression line and it is calculated by squaring them Equation (18).

(18) $$MSE=\frac{1}{n}\sum_{j=1}^{n}{\left(y_{j}-{\hat{y}}_{j}\right)}^{2}.$$

The root mean square error ( RMSE ) quantifies the standard deviation among prediction errors. It is defined as the square root of MSE , which is shown in Equation (19).

(19) $$RMSE=\sqrt{\frac{1}{n}\sum_{j=1}^{n}{\left(y_{j}-{\hat{y}}_{j}\right)}^{2}}.$$

Loss, also known as cross-entropy loss, is an effective approach for assessing classifier performance. It accomplishes this by examining the discrepancies between expected and actual probability distributions. It is worth noting that just 10% of classifiers can classify data, whereas decision tree classifiers have a log loss of 1.3515. In addition, cross-entropy may be calculated using P and Q probabilities. The importance of this methodology in data science cannot be emphasized since it provides a reliable and effective method for evaluating classifier performance.

H(P,Q) = −sum x inXP(x) × log(Q(x)).(20)

After applying various machine learning (ML) classifiers as shown in Table 5, the support vector machine (SVM) gives 92% accuracy, 72% precision, and 74% recall, although the main function of SVM is to correctly classify unseen data. By using the decision tree, the accuracy is 96%, precision 94%, and recall 91%; mostly, the use of the decision tree is for classification problem solving but it is also used in supervised learning techniques. Using Random Forest, the accuracy is 97%, precision 96%, and recall 93%; the random forest algorithm is used for classification as well as regression tasks, and mostly, it is applicable when handling large datasets. Using K-Nearest Neighbors (KNN) accuracy is 98%, precision 97%, and recall 95%; KNN is used to store complete the existing data and organizes a new data fact based on the resemblance. At last Naïve Bayes classifier predicts the maximum accuracy is 99.44%, precision 98.75%, and recall 96%. Naïve Bayes classifier is most used for the analysis of data, spam filtering data, and recommendation systems. It gives a fast response and easy implementation, but its biggest failure is the requirement for predictors to be self-determining.

The accuracy of blood pressure predictions is greatly enhanced by machine learning algorithms such as Naïve Bayes and K-Nearest Neighbors (K-NN), due to their distinct advantages and skills in evaluating and interpreting medical data:

- *Robust Predictions*: Our system leverages its combined strengths by employing multiple machine learning algorithms, including Naïve Bayes and K-NN. Naïve Bayes provides a robust probabilistic framework, while K-NN enhances the system’s ability to detect patterns and anomalies.
- *Complementary Strengths*: The different approaches of Naïve Bayes and K-NN complement each other, covering various aspects of data analysis and prediction. This synergy ensures that the predictions are accurate and resilient to variations and noise in the data.
- *Enhanced Model Performance*: Using an ensemble of algorithms (Naïve Bayes, K-NN, random forest, decision tree, and SVM) allows the system to cross-validate and refine predictions, further boosting overall accuracy and reliability.

Machine learning algorithms like Naïve Bayes and K-NN improve the accuracy of blood pressure predictions by leveraging their unique strengths in probabilistic modeling and instance-based learning. Their complementary capabilities ensure robust, accurate, and reliable predictions, which are crucial for effective real-time monitoring and management of chronic heart disease.

**Figure 4:**
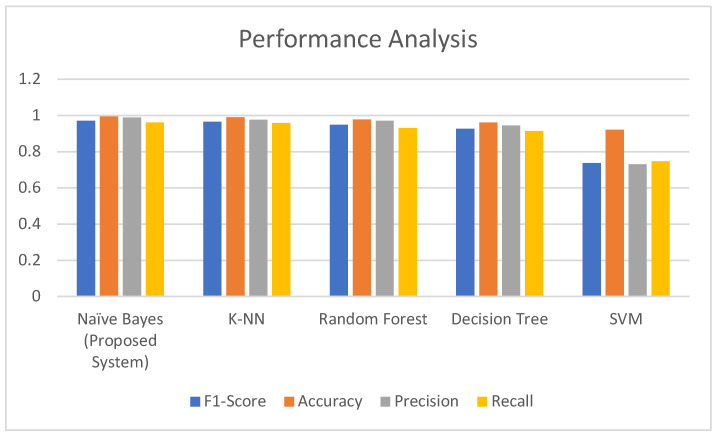
Assessment of the effectiveness of the proposed methodology compared to existing approaches.

Figure 4 compares the performance of our proposed IABPM model to existing machine-learning approaches. The study was conducted using four parameters: accuracy, precision, recall, and the F1 measure. Our suggested model beats all current techniques in terms of these parameters. The IABPM paradigm is intended to handle a range of issues by taking the output data from its consecutive levels as input. This feature offers particular benefits to each challenge. Our suggested technique also outperforms existing classical machine algorithms in terms of accuracy while minimizing mistakes. Additionally, processing time is lowered, as traditional machine algorithms are known to be time-consuming.

### 4.4. Early Prediction of Chronic Disease

The present article outlines a study that involves predicting the death rate using various parameters like R ^2^ score, R ^2^ adjusted, MSE, MAE, and RMSE. A comparison is used to assess the performance of several machine learning algorithms, including KNN, Random Forest, Decision Tree, and SVM. The outcomes of the proposed method, as presented in Table 6, demonstrate a lower error value than existing machine learning algorithms. Notably, the SVM performs the worst in this scenario. It is important to note that machine learning algorithms require structured data for prediction and detection processes. However, the proposed method is capable of working with unstructured data, making it smarter and more productive, with a lower error rate. This is a promising development that can lead to higher productivity in a shorter amount of time.

### 4.5. Performance Evaluation

**Figure 5:**
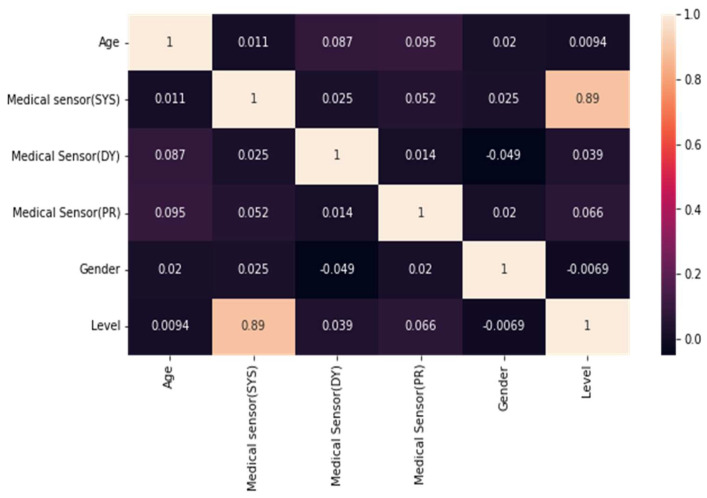
Correlation matrix using the database.

**Figure 6:**
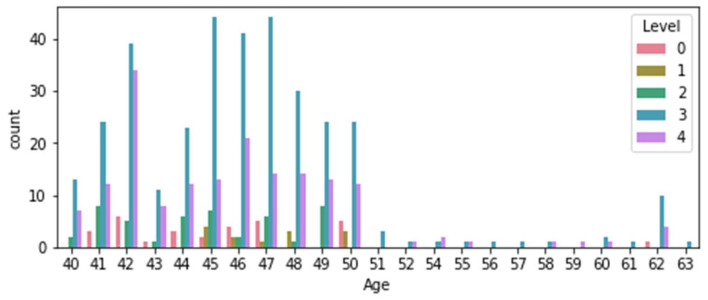
Number of patients per level.

Figure 5 displays the correlation matrix indicating the association between the target variable, level, and the other attributes. The Medical Sensor (SYS) is a cutting-edge diagnostic instrument that has been shown to have an impressive 89% correlation with level characteristics. Its outstanding effectiveness in recognizing patients categorized as level 3 or level 4 has received widespread recognition. According to Figure 6, the available statistics, adults aged 40 to 50 had the highest sensitivity to level 3 and level 4 classification. The data has been rigorously separated into training data (80%) and testing data (20%) to guarantee that the machine learning model achieves maximum accuracy. This strategy has proven to be extremely effective in lowering the proportion of patients classified as level 3 or level 4. The Medical Sensor (SYS) is a valuable tool for healthcare practitioners that has helped to improve patient care and overall outcomes in the medical area.

However, while reading the data, an automated serial number was assigned, resulting in entry_id being visible in Table 4, but not in the heatmap. This is a crucial factor that requires thorough attention and scrutiny when reviewing data to provide reliable and accurate outcomes.

### 4.6. Cost Effectiveness

To reduce healthcare costs and improve patient outcomes is necessary to utilize the IABPM module.

- Our IoT-Based Ambulatory Blood Pressure Monitoring System differs from existing systems by providing highly accurate, real-time data through advanced machine learning algorithms, and comprehensive monitoring capabilities.
- This innovation leads to better patient outcomes, early detection of CHD, and a significant reduction in healthcare costs.

Through continuous real-time monitoring, automated data collection and analysis, fewer hospital visits, early intervention through predictive analytics, resource optimization, and improved patient compliance, the IoT-Based Ambulatory Blood Pressure Monitoring System lowers healthcare costs. Compared to conventional techniques, these benefits result in considerable cost savings through more effective and efficient care of chronic heart disease (CHD).

The ROC-AUC curve serves as a valuable method for assessing the effectiveness of machine learning models in scenarios involving multiclass classification, particularly when the target variable is distributed across numerous classes. It is particularly effective in evaluating models that deal with two-class situations, such as predicting chronic illnesses. TPR (True Positive Rate), Recall, and Sensitivity are the most typical metrics used to evaluate model performance.

**Figure 7:**
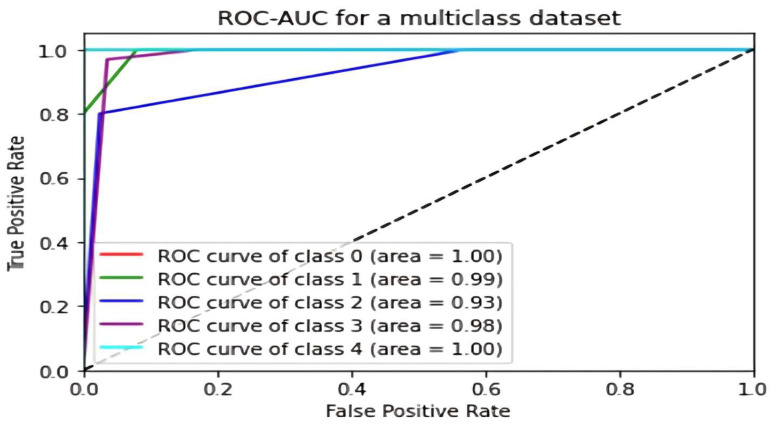
ROC-AUC curve for a multiclass dataset [26].

The ROC-AUC curve is a crucial tool for evaluating a model’s sensitivity and specificity, comparing its true positive rate (TPR) to its false positive rate (FPR), thereby assessing model performance. The ROC-AUC (Figure 7) curve facilitates the determination of the model’s efficacy by giving a visual depiction of its performance, with a higher area under the curve (AUC) indicating better performance. Additionally, using the TPR/Recall/Sensitivity equations, it is possible to effectively evaluate the model’s performance in a multiclass classification task involving chronic disorders.

### 4.7. Prediction and Testing

Machine learning approaches are chosen based on their capacity to reach peak performance in certain tasks, with an emphasis on striking a balance between accuracy, interpretability, and efficiency. The Naïve Bayes algorithm has developed as a viable machine learning tool, and it has been acknowledged for meeting physician needs. To assess the performance of the Naïve Bayes method, new data that corresponds to the training set is tested. Table 6 shows that the system correctly identifies crises that require immediate doctor consultation. Shalini Tai Meghe Hospital Research Center (SMHRC) Education in India., a highly respected medical research center, validated the algorithm’s performance.

The validation report connected to the text contains a full analysis of the algorithm’s performance, including its strengths and weaknesses. The study also provides a full explanation of the validation process, including methodology and testing results. The most appropriate machine learning approach must be chosen carefully, and it is determined by the specific situation at hand as well as the available resources. As a result, before deciding on a machine learning approach, it is critical to evaluate aspects such as data complexity, the needed degree of interpretability, and processing resources. The Naïve Bayes algorithm has shown promise in meeting the clinician criteria, and its performance has been assessed and confirmed by a respectable organization. The associated validation report contains a thorough examination of the algorithm’s performance, making it an invaluable resource for machine learning professionals and academics.

The current work presents a recommended approach for remote patient monitoring that includes the use of an intelligent automated blood pressure monitoring (IABPM) system. The IABPM system is intended to govern the operation of a medical sensor device according to a specified time interval set by medical practitioners. After collecting medical data, the medical sensor gadget transfers it to the cloud, where medical specialists can obtain it for further study.

**Figure 8:**
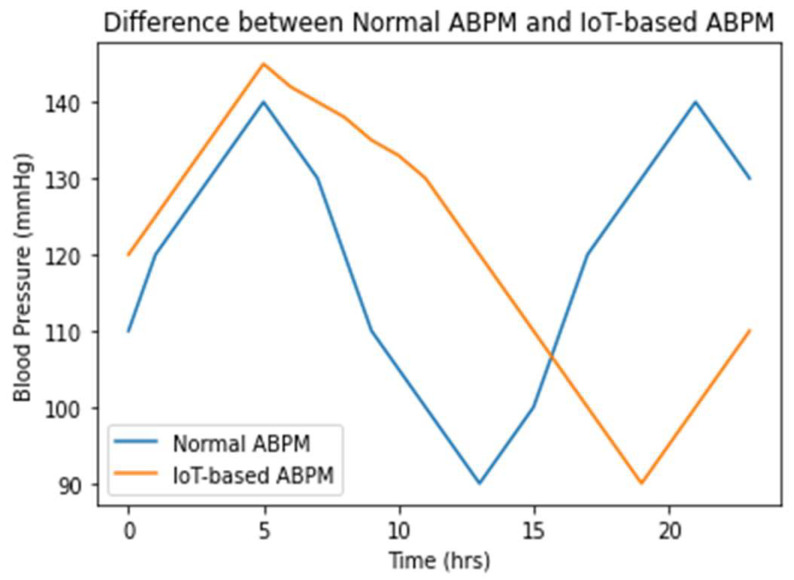
BP versus time difference between existing ABPM and IABPM.

The proposed approach for remote patient monitoring consists of an intelligent automated blood pressure monitoring (IABPM) system that continuously monitors blood pressure (BP) and pulse rate. The IABPM system is controlled by a timer, and a graphical comparison of BP values over 24 h is shown in Figure 8, comparing the IABPM system to the standard ABPM. The graph clearly illustrates that the IABPM system produces a normal curve, as indicated by the orange line.

### 4.8. Discussion

The IoT ambulatory blood pressure monitoring system (IABPM) is a new and innovative medical device that has been developed as an alternative to the current ambulatory blood pressure monitoring (ABPM) system. It is designed to offer real-time data monitoring, eliminate memory limitations, and provide an alert feature for patients with hypertension.

The IoT ambulatory blood pressure monitoring system (IABPM) is a promising medical technology that could revolutionize blood pressure monitoring. However, its real-time data monitoring and alarm feature may be challenging for hypertension patients. The lack of interfaces and limitations may also hinder its effectiveness in continuous monitoring, requiring careful consideration for both physicians and patients. Moreover, the IABPM system is discussed with the following parameters:

❖ Accuracy of Blood Pressure Prediction: The suggested Naïve Bayes model predicted blood pressure with an astounding 99.44% accuracy. This high degree of precision is critical for providing real-time intensive care to CHD patients, guaranteeing early detection and reaction to blood pressure variations.
❖ Machine Learning Algorithms: The study used machine learning techniques such as Naïve Bayes, K-NN, random forest, decision tree, and SVM to predict CHD early warning scores. This diversified group of algorithms strengthens the prediction model by accepting a variety of data types and enhancing overall accuracy.
❖ Game-Changing Impact: The IoT-Based Ambulatory Blood Pressure Monitoring System is seen as a game changer in healthcare. It overcomes the limits of previous BP monitoring systems by providing improved accuracy, continuous monitoring capabilities, and early identification of CHD anomalies. This technological development has the potential to drastically lower healthcare expenditures while improving patient outcomes.
❖ Revolutionizing Healthcare: By using this method, healthcare practitioners may make considerable progress toward improving patient outcomes and lowering the worldwide burden of CHD. Its extensive features provide precise and reliable diagnosis, which is critical for efficient CHD therapy and management.
❖ Potential for Revolutionizing Healthcare: Overall, the IoT-based ambulatory blood pressure monitoring system is a valuable tool for early identification and treatment of CHD. By offering accurate diagnosis, timely intervention, and ongoing monitoring, it has progressed in the healthcare industry, ultimately improving patient outcomes and care.

Significant implications for clinical practice stem from the Naïve Bayes model’s excellent prediction accuracy of blood pressure.

- It boosts CHD management with continuous monitoring and integration with predictive analytics, improves patient outcomes through early identification and tailored therapy, and increases the effectiveness of healthcare delivery by lowering diagnostic mistakes and sharing resources.
- Moreover, it supports preventative care activities and lowers hospital readmission rates, among other wider healthcare effects.
- The IoT-Based Ambulatory Blood Pressure Monitoring System has the potential to revolutionize the treatment of chronic heart disease and raise the standard of healthcare in general, as these advantages demonstrate.

In conclusion, the work carried out underscores the importance of early detection and accurate diagnosis in managing CHD and highlights the transformative impact of the IoT-based ambulatory blood pressure monitoring system in revolutionizing healthcare delivery.

## 5. Conclusions and Future Scope

The IoT Ambulatory Blood Pressure Monitoring System (IABPM) with deep learning testing could be beneficial for patients requiring homecare treatment for detecting, diagnosing, and managing CHD. Integration with other medical devices could provide remote medical care, while health insurance companies could use IABPM to reimburse treatment costs and attract more customers.

The National Crime Record Bureau reports a 53% increase in myocardial infarction mortality in India from 2014 to 2019. The IABPM, a real-time BP monitor, aims to reduce healthcare costs and detect chronic diseases. It offers advantages such as cost-effectiveness, real-time monitoring, long life, fast results, and a larger communication coverage area. The IoT technology saves enormous amounts of data in a cloud database, reducing hospital visits and improving patient quality of life. Its novel features offer significant information to healthcare experts and allow for personalized therapy for people suffering from this chronic ailment. One can make great progress in lowering the worldwide burden of CHD and improving patient outcomes by harnessing technology and predictive analytics. The proposed system achieves 99.44% accuracy which is greater than the previous methods.

The IABPM is a healthcare system that gives real-time monitoring and early warning scores to medical patients. It works with other medical equipment to enable patients to get care at home. IABPM can help health insurance companies reimburse treatment expenses by utilizing patient data logs stored on a cloud platform. The device can also assist in identifying people with white coat syndrome, a disorder characterized by elevated blood pressure readings in the doctor’s office and normal readings at home.

Address possible implementation obstacles with IoT-based ABPM systems, including data security and privacy, interoperability, and patient acceptability. Discuss ways to overcome these obstacles and make the most of this technology. Provide insights into future developments in IoT-based ABPM systems, such as advancements in sensor technology, integration with other health monitoring devices, and personalized predictive analytics. Summarize the potential impact of these technologies on revolutionizing CHD management and improving patient outcomes.

## 6. Patents

Patent Title: AN IOT BASED HEALTH MONITORING SYSTEM FOR CHRONIC DISEASES

Patent Number: 415982

Filed By:

(1) Prachi Gawande
(2) Yogesh Kale
(3) Shivam Sarve
(4) Shubhangi Rathkanthiwar

Filing Date: 29 December 2021

Publication Date: 29 December 2022

Description:

The main function of the IoT-based Blood Pressure Monitoring System (IABPM) device invention is to provide a flexible timer-setting facility. It also processes data in real time, enabling the acquisition of precise time-interval BP readings as prescribed by medical professionals for monitoring BP abnormalities in chronic patients. The connection of the invention with the research paper is to obtain all sample data using the invented IABPM device.

## Figures and Tables

**Table 1:** Hypertension stage range.

Blood Pressure Type	Upper Number	Lower Number
Normal	Less than 120 mmHg	Less than 80 mmHg
Elevated	120–129 mmHg	Less than 80 mmHg
First stage of hypertension	130–139 mmHg	80–89 mmHg
Second stage of hypertension	140–149 mmHg	90 mmHg or higher
Hypertension crisis Consult your physician immediately away.	Higher than 180 mmHg	Higher than 120 mmHg

**Table 2:** Comparison of previous work.

Authors and Reference No.	Algorithm/Method Used	Accuracy	Limitation
V. Tamilselvi et al. [10]	Arduino-Uno Board	>70%	There are no precise performance trials available for any of the patients.
Acharya and. Patil et al. [11]	Raspberry processor3	>65%	The system’s most critical issue is the lack of adequate interfaces for data visualization.
Gregoski et al. [12]	A mobile brightness and camera	<95%	If continuous heartbeat monitoring is necessary throughout the day without interfering with the patient’s mobility, the recommended approach is worthless.
Oresko et al. [13]	A mobile brightness and camera	<98%	The suggested prototype only identified cardiac rhythms in real time, rather than tracking heart rate (bpm) over time. As a result, it becomes challenging to detect cardiovascular disease.
Trivedi, and Cheeran et al. [14]	A system built using a smartphone and an Arduino.	<98%	This technology allows medical experts to remotely monitor and evaluate clinical data, as well as prescribe therapies.

**Table 3:** Systolic, diastolic, and pulse rate of a patient using IABPM.

Systolic Reading	Diastolic Reading	Pulse Rate Reading
124 mmHg	93 mm/Hg	77 bpm

**Table 4:** Pre-processing database [26].

Entry Id	Age	Med Sensor (SYS)	Med Sensor (DY)	Med Sensor (PR)	M/F	Level
1	45	125	88	84	0	1
2	48	126	76	75	0	1
3	46	127	89	89	0	1
4	45	128	85	80	0	1
5	46	129	94	79	0	1
-	-	-	-	-	-	-
.	.	.	.	.	.	.
.	.	.	.	.	.	.
.	.	.	.	.	.	.
595	42	156	92	82	1	3
596	46	199	99	83	1	4
597	62	210	100	99	1	4
598	41	165	70	98	1	3
599	44	168	69	102	1	3
600	48	144	78	76	1	3

(“-” indicates that middle no’s, for showing continuity).

**Table 5:** Performance analytics of the proposed system with other ML Techniques [26].

Classifier	F1-Score	Accuracy	Precision	Recall
Naïve Bayes (Proposed System)	0.9713	0.9944	0.9875	0.9600
K-NN	0.9642	0.9889	0.9765	0.9579
Random Forest	0.9480	0.9778	0.9692	0.9312
Decision Tree	0.9263	0.9611	0.9437	0.9137
SVM	0.7378	0.9200	0.7294	0.7467

**Table 6:** Result of Naïve Bayes proposed model.

Entry Id	Age	Med Sensor (SYS)	Med Sensor (DY)	Med Sensor (PR)	M/F
1	45	122	85	84	0
Result				You are Elevated	

## Data Availability

The data was shared with Shalini Tai Meghe Hospital and Research Center (SMHRC), as stated in the data availability statement. (Dataset Link: https://drive.google.com/file/d/11eCMmkD6DTwwrhFuSr2-zLDGRBwNWRrg/view?usp=sharing).
