# Correction: Kale et al. Revolutionizing Chronic Heart Disease Management: The Role of IoT-Based Ambulatory Blood Pressure Monitoring System. *Diagnostics* 2024, *14*, 1297

**DOI:** 10.3390/diagnostics16040519

**Published:** 2026-02-09

**Authors:** Yogesh Kale, Shubhangi Rathkanthiwar, Ganesh Yenurkar, Sandip Mal, Vincent O. Nyangaresi, Shailesh Kamble, Lalit Damahe, Nandkishor Bankar

**Affiliations:** 1 Department of Electronics and Telecommunication Engineering, Yeshwantrao Chavan College of Engineering, Wanadongri, Nagpur 441110, Maharashtra, India; ycceetyogesh@gmail.com; 2 Department of Electronics Engineering, Yeshwantrao Chavan College of Engineering, Wanadongri, Nagpur 441110, Maharashtra, India; 3 Department of Computer Technology, Yeshwantrao Chavan College of Engineering, Wanadongri, Nagpur 441110, Maharashtra, India; 4 School of Computing Science and Engineering, VIT Bhopal University, Bhopal 466114, Madhya Pradesh, India; sandip.mal@vitbhopal.ac.in; 5 Department of Computer Science and Engineering, Jaramogi Oginga Odinga University of Science & Technology, Bondo 40601, Kenya; 6 Department of Applied Electronics, Saveetha School of Engineering, SIMATS, Chennai 602105, Tamilnadu, India; 7 Department of Artificial Intelligence and Data Science, Indira Gandhi Delhi Technical University for Women, New Delhi 110006, Delhi, India; 8 Department of Computer Science and Engineering, Yeshwantrao Chavan College of Engineering, Wanadongri, Nagpur 441110, Maharashtra, India; 9 Department of Microbiology, Jawaharlal Nehru Medical College Sawangi Meghe, Wardha 442005, Maharashtra, India; drbankarnj28@gmail.com

## 1. Addition of Authors

The authors wish to correct the authorship of the original publication [1]. Yogesh Kale and Shubhangi Rathkanthiwar were not included as authors in the original publication. The corrected Author Contributions statement appears here.

Conceptualization, Y.K. and G.Y.; Supervision, S.R., S.M., V.O.N. and N.B.; Methodology, G.Y.; Visualization, G.Y. and L.D.; Writing—original draft, G.Y.; Software, G.Y. and L.D.; Validation, S.R., S.M. and N.B.; Writing—review and editing, V.O.N. and S.K.; Resources, Y.K. and N.B. All authors have read and agreed to the published version of the manuscript.

This authorship correction has been confirmed and approved by the affiliated institutions, Yeshwantrao Chavan College of Engineering (YCCE), Nagpur.

## 2. Missing Citation

There is a patent resulting from the work reported in this publication. However, the patent was not cited. The citation is: Gawande, P.D.; Kale, Y.S.; Sarve, S.G.; Rathkanthiwar, S.V. An IoT-Based Health Monitoring System for Chronic Diseases. Indian Patent Application No. 202121061417, 4 February 2022.

It has now been inserted in the captions of Figures 2, 3 and 7, and Tables 4 and 5, and should read:

**Figure 2.** IABPM flow diagram [26].

**Figure 3.** Circuit diagram of an IABPM [26].

**Table 4.** Pre-processing database [26].

**Table 5.** Performance analytics of the proposed system with other ML Techniques [26].

**Figure 7.** ROC-AUC curve for a multiclass dataset [26].

## 3. The Patent Section Has Been Added

Patent Title: AN IOT BASED HEALTH MONITORING SYSTEM FOR CHRONIC DISEASES

Patent Number: 415982

Filed By:

(1) Prachi Gawande
(2) Yogesh Kale
(3) Shivam Sarve
(4) Shubhangi Rathkanthiwar

Filing Date: 29 December 2021

Publication Date: 29 December 2022

Description:

The main function of the IoT-based Blood Pressure Monitoring System (IABPM) device invention is to provide a flexible timer-setting facility. It also processes data in real time, enabling the acquisition of precise time-interval BP readings as prescribed by medical professionals for monitoring BP abnormalities in chronic patients. The connection of the invention with the research paper is to obtain all sample data using the invented IABPM device.

## 4. The Conflicts of Interest Have Been Updated

Authors Yogesh Kale and Shubhangi Rathkanthiwar are co-inventors of a patent (Patent No. [415982]) describing the technology reported in this article.

## 5. Missing Acknowledgments

In the original publication, the Acknowledgments did not fully reflect all contributions. The corrected Acknowledgments section is as follows:

The authors would like to express their gratitude to N. J. Bankar, Department of Microbiology, JNMC, Wardha, for his valuable consent and the Shalini Tai Meghe Hospital Research Center Education (SMHRCE), Nagpur, for helping provide valid clinical data. Additionally, the authors express their thanks to Yogesh S. Kale, on behalf of Shubhangi V. Rathkanthiwar, Prachi D. Gawande, and Shivam G. Sarve, for their consent in using patent data, including figures, tables, and circuit diagrams, to support the technical details and claims presented in this research. (Indian Patent No. 415982, Application No. 202121061417).

The authors state that the scientific conclusions are unaffected. This correction was approved by the Academic Editor. The original publication has also been updated.

## References

[1] Kale Y., Rathkanthiwar S., Yenurkar G., Mal S., Nyangaresi V.O., Kamble S., Damahe L., Bankar N. (2024). Revolutionizing Chronic Heart Disease Management: The Role of IoT-Based Ambulatory Blood Pressure Monitoring System. Diagnostics.

